# Current Status of the Immunomodulation and Immunomediated Therapeutic Strategies for Multiple Sclerosis

**DOI:** 10.1155/2012/970789

**Published:** 2011-12-06

**Authors:** Shyi-Jou Chen, Yen-Ling Wang, Hueng-Chuen Fan, Wen-Tsung Lo, Chih-Chien Wang, Huey-Kang Sytwu

**Affiliations:** ^1^Department of Pediatrics, Tri-Service General Hospital, National Defense Medical Center, Taipei 114, Taiwan; ^2^Department of Microbiology and Immunology, National Defense Medical Center, Taipei 114, Taiwan; ^3^Center for Composite Tissue Allotransplantation, Chang Gung Memorial Hospital, Linkou, New Taipei City 333, Taiwan; ^4^Graduate Institute of Life Sciences, National Defense Medical Center, Taipei 114, Taiwan; ^5^Graduate Institute of Medical Sciences, National Defense Medical Center, Taipei 114, Taiwan

## Abstract

Multiple sclerosis (MS) is an autoimmune disease of the central nervous system, and CD4^+^ T cells form the core immunopathogenic cascade leading to chronic inflammation. Traditionally, Th1 cells (interferon-**γ**-producing CD4^+^ T cells) driven by interleukin 12 (IL12) were considered to be the encephalitogenic T cells in MS and experimental autoimmune encephalomyelitis (EAE), an animal model of MS. Currently, Th17 cells (Il17-producing CD4^+^ T cells) are considered to play a fundamental role in the immunopathogenesis of EAE. This paper highlights the growing evidence that Th17 cells play the core role in the complex adaptive immunity of EAE/MS and discusses the roles of the associated immune cells and cytokines. These constitute the modern immunological basis for the development of novel clinical and preclinical immunomodulatory therapies for MS discussed in this paper.

## 1. Multiple Sclerosis and Experimental Autoimmune Encephalomyelitis

Multiple sclerosis (MS) was initially identified in 1868 by Charcot. This disease often begins in young adulthood with intermittent episodes of neurological dysfunction, including visual impairment, ataxia, motor and sensory deficits, and bowel and bladder incontinence. These are attributable to recurrent inflammatory attacks on the white matter of the brain and spinal cord, which lead to the accumulation of perivascularly distributed inflammatory cells within the brain and spinal cord white matter [1].

Beeton et al. first established an animal model of MS in the 1930s, when they immunized monkeys with a central nervous system (CNS) homogenate to induce what is now known as experimental autoimmune encephalomyelitis (EAE) [2]. Since this pilot animal study, EAE has become the most accepted animal model of MS. In recent decades, pathogenic hypotheses have been investigated and novel therapeutic agents tested in this model in the fields of CNS inflammation and demyelination. Therefore, EAE provides a valuable tool for the investigation of the T-cell-dependent pathogenesis of autoimmune inflammation in the CNS and the orchestration of the autoimmune demyelinating inflammation in the CNS of MS patients. Mice and/or genetically modified mice have also been of fundamental value in the exploration of the complex pathogenesis of MS [3, 4]. EAE is undoubtedly the best animal model in which to study autoimmune diseases and particularly the demyelinating diseases of the CNS, such as MS [5].

## 2. Basic Immunopathogenic Mechanism and the Role of T Cells in EAE and MS

Myelin basic protein-(MBP)-specific T cells isolated from the peripheral lymphocytes of human individuals with MS and encephalitogenic T cells recovered from circulating autoreactive T cells of either immunized or naïve animals have shown that autoreactive T-cell lines that recognize the encephalitogenic part of MBP in vitro can be distinguished from an unprimed rat T-cell population. This confirms that autoreactive T cells play a central role in the pathology of MS [6–8]. EAE can also be induced by adoptively transferring an expanded population of myelin-reactive encephalitogenic CD4^+^ (T helper [Th]) cells, which allows the further dissection of the immunopathogenic potency of different encephalitogenic CD4^+^ cell populations [9].

In the 1990s, Mosmann and Coffman postulated that Th cells can be classified into two distinct subsets, Th1 and Th2. Th1 cells produce large quantities of interferon *γ* (IFN*γ*), driven by interleukin 12 (IL12), which promotes cellular immunity directed against intracellular pathogens. Alternatively, Th2 cells, which secrete IL4, IL5, IL13, and IL25, are essential in the destruction of extracellular parasites and the mediation of humoral immunity [10, 11]. Self-reactive Th1 clones derived in vitro are capable of adoptively transferring EAE to naïve recipients [12]. Increased levels of Th1 cytokines are particularly evident during EAE/MS relapse, whereas increased Th2 cytokines are found during remission in MS patients when compared with control levels [13]. Clinical and hematological symptoms are exacerbated in relapsing/remitting MS patients following the administration of IFN*γ*, and this is also observed in other Th1-type diseases, whereas it is less apparent in Th2 diseases [14, 15]. Th1 cells were earlier thought to be pathogenic T cells, whereas Th2 cells were thought to confer an anti-inflammatory potential, constituting protective T cells in both MS and EAE [16–19].

However, this clear-cut immunodysregulation of the Th1/Th2 balance in EAE and MS may be part of a hidden complex of interactions underlying EAE and MS [20]. The Th1-driven nature of the EAE/MS disease was challenged by the finding that IFN*γ*- and IFN*γ*-receptor-deficient mice, as well as mice that lack other molecules involved in Th1 differentiation, such as IL12p35, IL12 receptor *β*2 (IL12R*β*2), and IL18, were not protected from EAE, but instead were more susceptible to the disease [21–25]. Unexpectedly, mice deficient in IL12*α* (IL12p35), a component of the Th1 paradigm, are vulnerable to EAE. Similarly, IL12R*β*2-deficient mice develop more severe clinical manifestations of EAE, whereas IL12p40-deficient mice are resistant to EAE [23, 24, 26]. These discrepancies and conflicting data indicate that an imbalance in the Th1/Th2 milieu cannot explain the overall immunopathogenic mechanisms underlying EAE and MS.

## 3. Immunopathogenic Role of Th17 Cells and Cytokines in EAE/MS

p19, a novel cytokine heavy-chain homologue of the IL6 subfamily, was discovered as a computational sequence [27]. When the p19 chain is linked to the p40 chain, a subunit of IL12 (another subunit of the IL12 heterodimers is the p35 chain), it forms a novel cytokine designated IL23. Therefore, the deletion of IL12p40 will affect the functions of both IL12 and IL23. Cua and colleagues verified that Il23 but not Il12 is essential for the induction of EAE by generating Il23p19 knockout (KO) mice and comparing them with IL12p35 KO mice [28]. Furthermore, an IL17-producing T-cell subset, driven and expanded by IL23, can pathogenically induce EAE when adoptively transferred into naïve wild-type mice [29, 30]. These IL17-producing T cells were dramatically reduced in the CNS of IL23p19-deficient mice. Based on these studies, researchers confidently suggested that IL17-producing CD4^+^ T cells are a distinct and novel Th subset that exacerbates autoimmunity, and designated them Th17 cells [31, 32]. Th17 cells are a Th-cell subset distinct from Th1 and Th2 cells in terms of their differentiation, expansion, and effector functions [33, 34]. The discovery of Th17 cells further clarifies the cytokine profile of MS [35]. Recently, the levels of IL17 produced by MBP-stimulated peripheral blood cells obtained from MS patients or controls were shown to correlate with the active lesions in MS patients observed with magnetic resonance imaging (MRI) [36].

Like other Th subsets, the Th17 lineage is activated by a specific cytokine milieu. However, IL23 cannot produce Th 17 cells de novo from naïve T cells, and the IL23 receptor (IL23R) is not expressed on naïve T cells [37]. Transforming growth factor *β* (TGF*β*) upregulates IL23R expression, thereby conferring responsiveness to IL23, which confirms that TGF*β* is a critical cytokine in the commitment to Th17 expansion in vitro and in vivo [38]. In mice, TGF*β* together with IL6 can activate antigen-responsive naïve CD4^+^ T cells to develop into Th17 cells [39]. In humans, naïve CD4^+^ cells exposed to IL6, TGF*β*, and IL21 can develop into Th17 cells, and the production of IL23 plays a role in maintaining these Th17 cells [40, 41]. Altogether, Th17 cells require IL23, TGF*β*, IL6, and IL1 for their generation. Th17 cells produce IL17A and IL17F, which are upregulated in chronic lesions [42], and IL22, which is also involved in the pathogenesis of MS. Thus, Th17 cells are a recently discovered, unique Th lineage that produces a repertoire of signature cytokines, including IL17A, IL17F, IL21, and IL22, that are essential for the development of autoimmune diseases such as MS [43].

**Figure 1:**
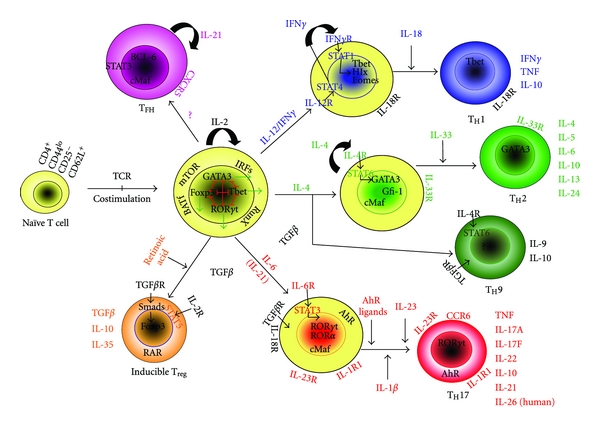
Current schedule of T-helper-cell differentiation. When naïve CD4^+^TCR*αβ* ^+^ T lymphocytes, classified by their low expression of CD44, absence of CD25, and high levels of CD62L, encounter their cognate antigens, they can differentiate into several previously identified effector subsets. It is likely that several “master” transcription factors, individually required for T-cell differentiation towards one of the end effector stages, are initially expressed upon engagement of the TCR with costimulatory receptors. Each transcription factor drives a specific set of genes required for lineage commitment and the expression of signature cytokines and negatively affects alternative pathways. However, the local microenvironment is the driving force that determines the outcome of the differentiation course. Th1 cells are established in the presence of IFN*γ* and IL12 and signaling via STAT1 and STAT4, resulting in the expression of the master transcription factor T bet. Th2 cells depend on IL4 and STAT6 for the increased expression of GATA3, whereas the simultaneous presence of TGF*β* results in the development of Th9 cells, utilizing an undefined master transcription factor. The presence of TGF*β*, with IL2 signaling via STAT5, is known to generate, at least in vitro, inducible Treg, which utilize FOXP3 like those Treg generated in the thymus. Again, it is TGF*β* in combination with IL6 signaling via STAT3 that drives the expression of ROR*γ*t, resulting in the differentiation of Th17 cells. However, the initiation of the developmental program of these T helper subsets may not be completed in the presence of only these driving cytokines. Several additional factors may be required for their subsequent functional maturation or may be responsible for the fine tuning of their effector phases. Several of these factors are indicated, together with the characteristic cytokine profiles of each subset (adapted from [54]).

The discovery of transcription factors that are key regulators of the cytokine expression required to launch lineage-specific transcriptional programs has greatly extended our understanding of Th-cell lineage commitment [44]. It has been shown that T-bet and STAT4 program the commitment of the Th1 lineage and Th1 cytokine production [45], whereas GATA-binding protein 3 (GATA3) and STAT6 drive Th2 population expansion and Th2 cytokine production [46, 47]. The T-bet and STAT4 (necessary for Th1 differentiation) transcription factors are important in the differentiation of autoimmune T cells in the EAE model [48], and T-bet- and STAT4-deficient mice are resistant to EAE. However, these transcription factors do not mediate the induction of Th17 cells. Instead, in a unique inductive milieu, Th17 differentiation is driven by distinct transcription factors: retinoic acid receptor-related orphan receptor-*γ*t (Ror*γ*t) and Ror*α* [33, 34]. Stat3 deletion in T cells also prevents autoimmune uveitis and EAE and increases the expression of IL10 and forkhead box P3 (FoxP3) [49], and the expression of FoxP3 programs the development and functions of Treg cells [50]. In humans, IL23 and IL1*β* also induce the development of Th17 cells expressing IL17A, IL17F, IL22, IL26, IFN*γ*, the chemokine CCL20, and the transcription factor ROR*γ* [51–53], as illustrated in Figure 1 (adapted from Hirota et al. [54]).

 Recent microarray studies of lesions in MS patients demonstrated an increased expression of IL17, confirming that Th17 cells play an important role in the development of inflammation and demyelination and in the eventual damage of the CNS. IL17 is a recently described cytokine produced in humans almost exclusively by activated memory T cells and can induce the production of proinflammatory cytokines and chemokines from parenchymal cells and macrophages. Patients with MS have greater numbers of IL17-mRNA-expressing mononuclear cells in the cerebrospinal fluid (CSF) than in the blood. Previously, no increase in the numbers and expression of IL17 mRNA by mononuclear cells isolated from the CSF was observed in patients with MS, but higher levels of IL17 mRNA were observed in the CSF than in the blood, with the highest levels in the blood detected during clinical exacerbations [56]. These data confirm the pivotal role of IL17 in MS both peripherally and centrally.

## 4. Recruited and Residential Innate Immune Cells in EAE and MS

Myelin is expressed in the circulation, and other CNS antigens are thought to be expressed in the cervical lymph nodes, which can trigger the conversion of autoaggressive myelin-reactive T cells to pathogenic T cells. Adhesion molecules, the integrins, allow these myelin-reactive T cells to penetrate the blood-brain barrier (BBB) under inflammatory conditions, and in this way, activated and memory T cells can enter the CNS [57]. Autoaggressive myelin-reactive T cells migrate into the CNS, where they recognize their cognate target antigens, and the movement of antigen-presenting cells (APCs) into the CNS is essential for lymphocyte reactivation within the CNS compartment and the initiation of the inflammatory cascade in the development of EAE [58]. Subsequently, inflammatory and immune cells, such as granulocytes and macrophages, are attracted into the CNS parenchyma, where they mediate tissue inflammation, leading to demyelination and tissue damage [59].

The brain was formerly considered an immunoprivileged organ, but this perspective has been revised in the last two decades [60]. Today, we understand that any damage to the CNS can activate immune cells in situ in the CNS, particularly microglial cells. Deshpande et al. demonstrated the transient inactivation of microglial cells via a cell-specific deficiency of CD40 expression, indicating that microglial cells are crucial for maintaining the autoimmune responses in the CNS [61]. The major histocompatibility complex (MHC, also known as “human leukocyte antigens” in humans) class II molecules are only displayed on specialized APCs (e.g., dendritic cells [DCs], B cells, and macrophages), whereas MHC class I molecules are expressed by all cells in the inflammatory milieu of the CNS [62]. Microglial cells upregulate the expression of MHC and costimulatory molecules to initiate the generation and maintenance of the inflammatory milieu. DCs seem to play a critical role in antigen presentation to invading T cells and in the release of cytokines and chemokines, thereby guiding the entry of monocytes, lymphocytes, and cells with a phenotype similar to that of DCs into the lesion [63].

Th cells recruit macrophages, which release proinflammatory cytokines and destructive molecules (such as nitric oxide [NO], IL1, IL6, tumor necrosis factor *α* [TNF*α*], and matrix metalloproteinases (MMPs]), and CD8^+^ T cells also directly attack MHC class I-expressing cells, such as oligodendrocytes and neurons [64, 65]. The secretion of destructive molecules, such as NO and TNF*α*, and the degradation of myelin are consequences of this cascade. TNF receptor 1 (TNFR1) but not TNFR2 signaling is critical for demyelination and the limitation of T-cell responses during immune-mediated CNS disease [66]. This complicated process triggers the recruitment of innate immune cells, generally consisting of T cells, macrophages, and microglia, which in turn mediate demyelination, axonal damage, and lesions.

## 5. Th17 and Immune Cells In Situ

In autopsy samples from MS patients, the expression of IL17 is evident in perivascular lymphocytes and in astrocytes and oligodendrocytes located in the active areas of CNS lesions. IL17R is also identifiable in acute and chronic MS plaques of patients with MS, suggesting the enrichment of Th17 and CD8^+^ T cells in active MS lesions, and confirming an important role for IL17 in the pathogenesis of MS [67]. Th17 cells are identified by their expression of IL23R and the memory T-cell marker CD45RO in situ. Other markers that have been investigated including the chemokine receptor, CCR6, and RORC variant 2, which is a central transcription factor for Th17-cell development [42, 68]. Microarray analysis of MS lesions has also demonstrated increased transcripts of genes encoding inflammatory cytokines, particularly IL6, IL17, and IFN*γ* and associated downstream pathways [56]. A significant increase in IL23 mRNA and protein expression is found in lesion tissues compared with nonlesion tissues. Activated macrophages/microglia have been shown to be important sources of IL23p19 in active and chronically active MS lesions. IL23p19-expressing mature DCs are preferentially located in the perivascular cuffs of active lesions. This data on the expression of IL23p19 in MS lesions improves our understanding of the pathogenesis of MS [69].

There is also evidence that MS endothelial cells express high levels of IL17R and are more permeable to IL17 than are non-MS endothelial cells. Perivascular DCs also express high levels of granzyme B in inflammatory lesions, polarizing naïve CD4^+^ T cells into Th17 cells. These Th17 cells transmigrate efficiently across BBB endothelial cells (BBB-ECs), leading to the destruction of human neurons and initiating CNS inflammation through Th-cell recruitment [70]. Similarly, the expression of IL17R and IL22R on BBB-ECs has been examined in MS lesions, and IL17 and IL22 have been shown to disrupt BBB tight junctions in vitro and in vivo. IL6 transsignaling may also play a role in the autoimmune inflammation of the CNS, mainly by regulating the early expression of adhesion molecules, possibly via cellular networks at the BBB [71]. Ifergan et al. demonstrated that a subset of CD14^+^ monocytes migrate across the inflamed human BBB and differentiate into CD83^+^CD209^+^ DCs under the influence of BBB-secreted TGF*β* and granulocyte-macrophage colony-stimulating factor (GM-CSF). These DCs can produce IL12p70, TGF*β*, and IL6 and promote the proliferation and expansion of distinct populations of Th1 and Th17 cells. The abundance of such DCs in situ is strongly associated with microvascular BBB-ECs within acute MS lesions and with a significant number of Th17 cells in the perivascular infiltrate [72].

Astrocytes play significant physiological roles in CNS homeostasis and act as a bridge between the CNS and the immune system. Astrocytes also contribute to the complex interactions during CNS inflammation. IL17 functions in a synergistic manner with IL6 to induce IL6 expression in astrocytes. Astrocytes upregulate the expression of IL17 and IFN*γ* genes and proteins in T cells, which is consistent with the astrocytes' capacity to express IL23 subunit p19 and the common IL12/IL23 subunit p40, but not IL12 subunit p35 when these two cell types are cocultured [73]. Das Sarma et al. demonstrated increased IL17RA expression in the CNS of mice with EAE and the constitutive expression of functional IL17RA in mouse CNS tissues. They also identified the expression of IL17RA in both astrocytes and microglia in vitro. In that study, the secretion of the chemokines Mcp1, Mcp5, Mip2, and CxcL1 was upregulated in these cells, suggesting that the upregulation of chemokines by glial cells is the result of IL17A signaling through constitutively expressed IL17RA [74].

Ma et al. demonstrated that the suppressor of cytokine signaling 3 (Socs3) participates in IL17 functions in the CNS as a negative feedback regulator, using mouse models of Socs3 small interfering RNA (siRNA) knockdown and Socs3 deletion. These mice with loss of Socs3 function showed enhanced IL17 and IL6 signaling in astrocytes via the activation of the NF-*κ*B and Mapk pathways, indicating that astrocytes can act as a target of Th17 cells and IL17 in the CNS [75]. Similarly, Kang et al. constructed specific deletion mutants of Act1, a critical component required for IL17 signaling, in mice with EAE to examine CNS inflammation in endothelial cells, macrophages, microglia, and the neuroectoderm (neurons, astrocytes, and oligodendrocytes). In these Act1-deficient mice, Th17 cells showed normal infiltration into the CNS but failed to recruit lymphocytes, neutrophils, and macrophages. Therefore, astrocytes are critical in IL17–Act1-mediated leukocyte recruitment during EAE [76].

Interestingly, Merkler et al. demonstrated that macrophages respond to the Th1 milieu and neutrophils respond to Th17 cytokines in a marmoset monkey model of EAE. They also showed dense accumulations of T and B lymphocytes, MHC-II-expressing macrophages/microglia, and early activated macrophages at the sites of perivascular and parenchymal lesions in the neocortex and subcortical white matter, indicating that the inflammatory response, especially macrophage and microglia activation, may be regulated differently in the gray matter areas of the primate brain [77].

In summary, DCs in the peripheral tissues and microglia in the CNS are responsible for cytokine polarization and the expansion of Th17 cells. The complex interactions of Th17 cells with different DCs, such as microglia, astrocytes, and peripheral DCs (including neutrophils and macrophages), all contribute to the immunopathogenesis of EAE and MS.

## 6. Reciprocal Interactions of Cytokines on Th Subsets in EAE/MS

IL1R KO mice have impaired Th17 cells and are protected from EAE [78], and IL1*β* increases the susceptibility to and progression of relapse onset in MS [79], implying a role for IL1*β* in the development of EAE and MS. EAE was abolished by a virus-expressing IL4 but not by a virus-expressing IL10 in chronic relapsing EAE. Therefore, the cytokine environment was converted from a disease-promoting IL23-producing condition to a disease-limiting IL4-producing condition by the local expression of IL4 from a Herpes simplex virus vector delivered to the brain [80]. Moreover, the increased expression of IL4 in glial cells was associated with the reduced severity of EAE [81], suggesting that the upregulation of Th2 cytokines inhibits the propagation of the inflammation of EAE/MS by encephalitogenic Th17 cells. CD4^+^CD25^+^Foxp3^+^ T cells, well-known regulatory T cells (Tregs), retain the potential to inhibit the autoimmune response, and protect against inflammatory injury. TGF*β* is a key cytokine in the generation of Tregs. Tregs are not only primarily involved in the regulation of Th17 cells but can also regulate the functions of Th1/Th2 cells [82]. A distinction has been drawn between the generation of pathogenic Th17 cells that induce autoimmunity and the generation of Tregs that inhibit autoimmune tissue injury [39].

Although EAE was once considered a classical Th1 disease, it has been proposed that it is predominantly Th17 driven. Recently, Singh et al. demonstrated that the overexpression of IL17 in T cells did not exacerbate EAE. Moreover, genetic and antibody studies have indicated that the absence of IL17A or IL17F does not reduce the incidence or severity of EAE. The collective findings of IL17 and IFN*γ* studies indicate that their roles may depend on the nature of the immune response and that the IL17 that occurs in the brain may overcome the inhibitory effect of IFN*γ*, which generally prevents inflammation at that site [83]. When pure Th17 cells from myelin oligodendrocyte glycoprotein-(MOG-) immunized mice, polarized with TGF*β* to deplete any IFN*γ* production, are adoptively transferred to mice, they do not induce EAE, suggesting that the reciprocal interactions among Th17-related cytokines enrol and activate the involvement of associated immune cells. Interestingly, when Th17 cells are combined with Th1 cells, they can fully induce EAE disease [84]. Liu et al. also demonstrated that the loss of STAT3 by Th cells results in an intrinsic developmental defect that renders STAT3^−/−^ mice resistant to CNS inflammatory diseases. STAT3 is required for the production of IL17 by Th17 cells, the generation of double positive T cells expressing IL17 and IFN*γ*, and T cell trafficking into CNS tissues. This suggests that STAT3 may be a therapeutic target for modulating CNS autoimmune diseases, and that Th1 cells can facilitate the entrance of Th17 cells into the CNS during EAE [85].

An encephalitogenic Th1 cell line that induces the recruitment of host Th17 cells to the CNS during the initiation of EAE has been reported [49]. Stromnes et al. showed significant differences in the regulation of inflammation in the brain and spinal cord, depending on different Th17/Th1 ratios, by demonstrating that specific T-cell populations targeting different myelin epitopes are characterized by different Th17/Th1 ratios in EAE [86]. Therefore, Th1 cells have the potential to reciprocally regulate Th17 cells during EAE.

IL21 is a type I four-*α*-helix bundle cytokine that belongs to the IL2 family and functions as a “growth hormone”-like cytokine. After the antigen-responsive differentiation phase, Th17 cells enter the amplification stage, and IL21 plays a pivotal role in the expansion and differentiation of the Th17 lineage, providing an autocrine and paracrine stimulus for Th17 cells [41, 87]. During clonal expansion, IL21 also promotes IL23R expression in differentiated Th17 cells, which plays an important role in the stabilization of the Th17 lineage in the presence of IL23 [88]. Although no effects were observed when Il21 was administered after EAE progression, the administration of IL21 boosted natural killer (NK) cell functions before the induction of EAE, including the secretion of Ifn*γ*. Therefore, IL21, by affecting NK cells, has various effects during the initiation and progression of EAE [89].

Alternatively, IL27, an IL12/IL23 family member, is a negative regulator of Th17 cell differentiation and can prevent inflammatory demyelination in the EAE model [44]. IL27 drives the expansion and differentiation of IL10-producing Tr1 cells by inducing the expression of three key molecules: the transcription factor c-MAF, the cytokine IL21, and ICOS. Moreover, IL27-driven c-MAF expression transactivates the production of IL21, which acts as an autocrine growth factor for the expansion and/or maintenance of IL27-induced Tr1 cells. ICOS also promotes IL27-driven Tr1 cells. Each of these elements is essential, because the loss of c-MAF, IL21 signaling, or ICOS reduces the frequency of IL27-induced differentiation of Tr1 cells (Figure 1) [90]. Exacerbation of EAE was demonstrated in IL27-deficient mice, and interestingly, Il27-treated mice had markedly reduced CNS inflammatory infiltration, indicating the downregulation of Th17 phenomena [91].

Recently, a novel effector T-cell subset, Th9 cells, has been identified, and the ability of this T-cell subset to induce EAE is currently being investigated. Jäger et al. generated Mog-specific Th17, Th1, Th2, and Th9 cells in vitro to directly characterize their encephalitogenic potency after their adoptive transfer. They found that Mog-specific Th1, Th17, and Th9 cells, but not Th2 cells, induce EAE. Interestingly, each T-cell subset induced disease in a distinct pathological manner, suggesting that the different effector Th subsets that induce EAE do so differently and implying that the pathological heterogeneity in MS lesions might be partly attributable to various characteristics of myelin-reactive effector T cells [92]. The authors also suggested that MS might be a disease caused by multiple distinct myelin-reactive effector cells. The disease induced by Th17 cells in some animals exhibited symptoms atypical of EAE, including ataxia, severe imbalance, and weight loss associated with high mortality. Some animals had a mixture of atypical and typical EAE symptoms. When cells were recovered from the CNS, it appeared that the transferred Th9 cells produced IFN*γ*. The identities of the other cell populations did not seem to drift after their in vivo transfer [93].

Nowak et al. recently demonstrated that like other T cells cultured in the presence of TGF*β*, Th17 cells produce IL9. Th17 cells generated in vitro with IL6 and TGF*β* and ex vivo-purified Th17 cells both produced IL9. Data show that IL9 neutralization and IL9R deficiency attenuate the disease, and this correlated with reductions in Th17 cells and IL6-producing macrophages in the CNS. These authors also confirmed the role of IL9 in the development and progression of EAE and implicated Il9 as a Th17-derived cytokine that contributes to inflammatory disease [94].

Together, Th2 cells, Tr1 cells, and Tregs exert repressive effects on Th17 cells, and Th9 cells have a stimulatory effect on Th17 cells, suppressing EAE and MS. However, Th1 cells play dual roles in EAE.

## 7. Clinical Applications, Limitations, and the Future of Immunomediated Therapies for MS

Our understanding of the pathophysiology and neurodegenerative processes of MS has led to the development of novel therapeutic strategies. Since the early 1990s, disease-modifying drugs have been introduced for the selective management of MS, including IFN*β* and glatiramer acetate (GA), which have become the standard treatment for relapsing/remitting MS [95]. Most recommendations previously made by the Multiple Sclerosis Therapy Consensus Group (MSTCG) on the use of disease-modifying drug therapies remain valid [96, 97]. Hermmer and Hartung have published an apparent review of the development of rational therapies in MS [98]. Therefore, we will discuss four domains of novel immunomediated therapeutics used for MS and their current status.

The first domain includes immunosuppressive agents, such as mitoxantrone, laquinimod (ABR-215062), cladribine (Mylinax ), and teriflunomide (probably via the suppression of TNF*α* and IL2 production). The second domain includes immunomodulatory agents: (1) cytokine inhibitors such as IFN*β*; (2) agents that deplete specific immune cell subsets, such as alemtuzumab (a human monoclonal antibody [mAb] that targets CD52 expressed by T and B cells, producing long-term T-cell depletion) [99, 100] and rituximab (which targets CD20 to deplete human B cells) [99, 101]; (3) agents that selectively block coreceptors and costimulators, such as daclizumab (an anti-CD25 mAb that inhibits activated T cells and induces regulatory immune cells) [102]. The third domain involves the development of migration-modifying therapies: (1) agents that affect adhesion molecules, such as natalizumab (an mAb that blocks very late antigen 4 [VLA-4]) and (2) sphingosine 1-phosphate receptor (S1PR) agonists: fingolimod (FTY720). The fourth domain includes neuroprotective agents associated with immunomodulation, including broad-spectrum immunomodulators such as statins, PPAR agonists (e.g., pioglitazone, gemfibrozil), the sex hormone estriol (E3), fumarate, minocycline, and erythropoietin (EPO), all of which have been effective in the treatment of both EAE, and MS. IFN*β* has been clinically introduced to treat patients with MS based on its ability to shift a Th1-mediated response to a Th2-mediated response [92]. However, microarray studies have indicated that a number of genes in patients with MS are upregulated by the cytokines associated with the differentiation of cells into Th1 lymphocytes rather than into Th2 lymphocytes, suggesting that this shift may not be the only therapeutic mechanism of IFN*β* in MS [103]. IFN*β* therapy also reduces IL23 mRNA levels [104]. IFN*β* inhibits human Th17 cell differentiation, so the Th17 axis could be another target of IFN*β* therapy [105]. IFN*β*-mediated IL27 production by innate immune cells has been shown to play a critical role in the immunoregulatory role of IFN*β* in EAE by inhibiting Th17 cells in EAE mice and MS patients [91, 106, 107]. Besides, Galligan et al. evidence further that IFN*β*(−/−) mice exhibited an earlier disease onset and a more rapid progression of EAE compared to IFN*β*(+/+) mice of EAE and IFN*β*(−/−) mice of EAE had increased numbers of CD11b(+) leukocytes infiltrating affected brains and an increased percentage of Th17 cells in the CNS with augmentation of autoreactive T cells,suggesting that IFN-*β* acts to suppress the production of autoimmune-inducing Th17 cells during the development of disease as well as modulating proinflammatory [108]. In addition, the therapeutic effect of IFN*β* is probably attributable to the induction of the regulatory cytokine IL10 [104]. Furthermore, Axtell et al. design a delicate study to further clarify the role of IFN*β* in MS/EAE [109]. Likewise, They demonstrate that IFN*β* was effective in reducing EAE symptoms transferred by Th1 cells transfer but exacerbated disease by Th17 cells transfer and effective treatment of IFN*β* in Th1-induced EAE correlated with augmented IL10 production; differently, in Th17-induced EAE, the amount of IL10 was unaffected by treatment of IFN*β*. Likewise, a high IL17F level in the serum of people with RRMS is associated with fail of IFN*β* therapy. This characteristic of IFN*β* might contribute to explore some logical biomarkers for predictive assessment of the response to a popular therapy for MS [109, 110]. Although, B cells may have a dual role in the pathogenesis of MS that they contribute to the induction of the autoimmune response but also mediate the resolution of the CNS inflammatory infiltrate [111, 112]. However, Ramgolam et al. demonstrate further that supernatants transferred from IFN*β*-1b-treated B cells inhibited Th17 cell differentiation, as they suppressed gene expression of the RORC and IL-17A and secretion of IL-17A. Likewise, IFN*β*-1b also induces B cells' IL-10 secretion which may mediate their regulatory potent [113]. Thus, IFN*β*-1b exerts its therapeutic effects at least in part by targeting B cells' functions that contribute to the autoimmune pathogenesis of RR MS, which may uncover extra mechanisms of the B-cell contribution to the autoimmune effects and provide novel targets for future selective treatment of MS [113].

Glatiramer acetate (GA; Copaxone; copolymer 1) exerts a clinical response in MS patients via its modulation of IFN*γ* and IL4 by reducing the expression of IFN*γ* and ensuring the stable expression of IL4 in anti-CD3/CD28-stimulated peripheral blood mononuclear cells (PBMCs) [114]. Moreover, GA enhances the suppressive effects of Tregs in both EAE and MS [115, 116]. Studies of human DCs have shown that GA modulates the production of inflammatory mediators without affecting DC maturation or immunostimulatory potential. DCs exposed to GA secrete low levels of the Th1-polarizing factor IL12p70 in response to lipopolysaccharide and triggering of the CD40 ligand [117]. Human DCs exposed to GA also induce IL4-secreting effector Th2 cells and increase their expression of IL10 [118]. These results show that APCs, including DCs, are essential for the GA-mediated shift in Th-cell phenotypes and indicate that DCs are an important target of the immunomodulatory effects of GA.

Patients with MS show a threefold to fourfold increase in the expression of the *α*4 subunit of the integrin VLA-4, which is normally expressed on activated lymphocytes, monocytes, and other cell types in the CSF and circulation [119]. Elovaara et al. confirmed that methylprednisolone reduces the adhesion molecules in the blood and CSF in patients with MS [120], implying that targeting leukocyte trafficking may be a possible therapeutic strategy for MS [121]. Therefore, natalizumab, a humanized mAb directed against the VLA-4 adhesion complex, has been introduced into the treatment of MS and reduces the risk of sustained progression of disability and the rate of clinical relapse in patients with relapsing MS [122]. However, during clinical trials, two natalizumab-treated MS patients developed progressive multifocal leukoencephalopathy (PML), which resulted in the voluntary removal of the drug from the market in February 2005 [123, 124]. A retrospective safety evaluation was subsequently conducted, and natalizumab was consequently returned to the market as a monotherapy in July 2006 for the treatment of relapsing MS; however, there were 111 cases of PML reported subsequently in natalizumab-treated MS patients as of April 2011 [125]. More evidently, the risk of developing PML for a MS patient on natalizumab (Tysabri) is almost 100 times higher if the patient (1) has been taking the drug for more than two years, (2) has a prior history of immunosuppressant use, and (3) tests positive for antibodies to the JC virus [126], compared to a patient with none of these three risk factors [127]. Instead, there is currently no convincing evidence that natalizumab-associated PML is restricted to combination therapy with other disease-modifying or immunosuppressive agents [128]. Nevertheless, natalizumab use must be restricted to the indicated patients.

Mitoxantrone, a cytotoxic drug with immunomodulatory properties, is used to treat progressive forms of MS [129]. Mitoxantrone increases the ex vivo production of the Th2 cytokines IL4 and IL5, but with no significant changes in IFN*γ*, TNF*α*, IL10, or IL17 expression by PBMCs or CD4^+^ T cells, indicating that the immunomodulation afforded by mitoxantrone treatment in MS acts through the enhancement of Th2-type cytokines [130].

**Figure 2:**
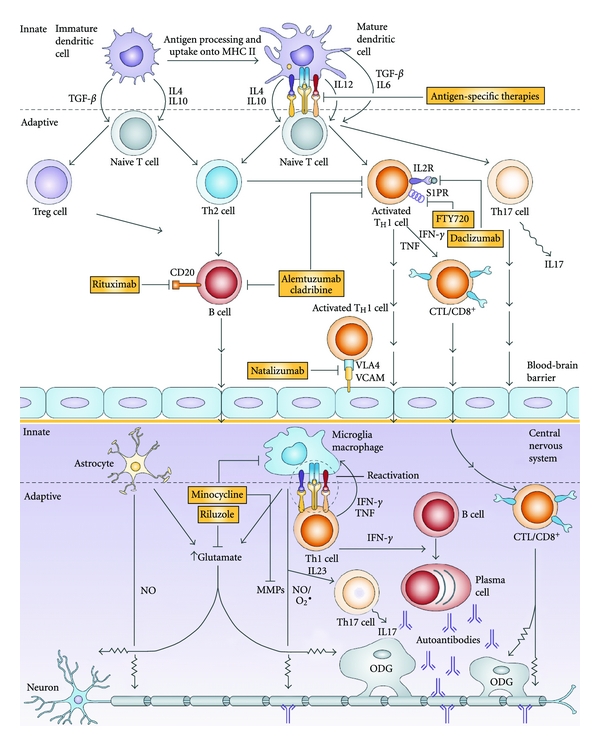
Multiple sclerosis immunopathogenesis and therapeutic targets. Immature dendritic cells (DCs) are central players in the innate immune response and are involved in the maintenance of peripheral tolerance by promoting the suppressor Treg and anti-inflammatory Th2-cell responses. Abnormally activated (mature) antigen-presenting DCs can be found in patients with multiple sclerosis (MS). This activation results in the increased production of proinflammatory cytokines, which lead to the aberrant activation of Th1 and Th17 proinflammatory responses. Activated encephalitogenic adaptive immune effectors (such as Th1 cells, Th17 cells, CD8^+^ cells, and B cells) express surface molecules that allow them to penetrate the blood-brain barrier and to enter the central nervous system (CNS). The presence of autoreactive immune effectors, together with abnormally activated CNS astrocytes and microglia, leads to the increased production of reactive oxygen species, excitotoxicity, autoantibody production, and direct cytotoxicity, which are all involved in the demyelination and axonal and neuronal damage that is present in patients with MS. Potential therapeutic interventions at different levels of the immunopathological cascade are shown in the filled yellow boxes (cytotoxic T lymphocytes [CTL]; interferon *γ* [IFN*γ*]; IL2 receptor [IL2R]; major histocompatibility complex class II [MHC II]; matrix metalloproteinases [MMPs]; nitric oxide [NO]; oligodendrocyte [ODG]; sphingosine 1-phosphate receptor [S1PR]; transforming growth factor *β* [TGF*β*]; tumor necrosis factor [TNF]; regulatory T cells [Treg]; vascular cellular adhesion molecule 1 [VCAM1]; very late antigen 4 [VLA-4] (This figure was adapted and partly revised from [55].).

Currently, a head-to-head race for approval had initially developed between two under spotlight oral immunomodulatory agents—fingolimod and cladribine (Figure 2) [131]. Fingolimod (FTY720/Gilenya, Novartis), an S1PR modulator [132], is under the spotlight because it has completed phase III trials [133] and has been approved by the US Food and Drug Administration as the first oral, first-line treatment for relapsing MS [134, 135]. S1PR is mainly expressed by immune cells, neuronal cells, endothelial cells, and smooth muscle cells [136–139]. The key roles of S1PR in angiogenesis, neurogenesis, and the regulation of immune cell trafficking, endothelial barrier function, and vascular tone were demonstrated with the genetic deletion of S1pr in a murine model [140–142]. The immunomodulatory effect of fingolimod acts in two pathways. In one pathway, it inhibits the function of S1PR, which facilitates the CC-chemokine receptor 7-(CCR7-) mediated retention of lymphocytes in the lymph nodes, including naïve T cells and central memory T cells, but not effective memory T cells. This significantly reduces the infiltration of inflammatory cells into the CNS [143, 144] and reduces the numbers of autoreactive Th17 cells that are recirculating via the lymph and blood to the CNS [145–147]. The second pathway prohibits neuroinflammation via the modulation of the S1PR1 expressed on oligodendrocytes, neurons, astrocytes, and microglia [76, 148, 149]. Another oral immunomodulatory drug Cladribine (2-chlorodeoxyadenosine) is a synthetic chlorinated deoxyadenosine analog [150] that is activated by intracellular phosphorylation in specific cell types, resulting in preferential and sustained reduction of peripheral T and B lymphocytes, mimicking the immune-deficient status of hereditary adenosine deaminase deficiency [151]. Orally administered cladribine shows significantly efficacy in patients with RR-MS [152]. Relative to placebo, oral cladribine reduces relapses by 55–58% and has an impact on disability progression and all MRI outcome markers in patients with RR-MS [152–154]. Nevertheless, to exactly weight the benefits of both novel immunomodultory agents against the potential risks is necessary and must be monitored continually.

 These advances in identifying unique therapeutic targets for MS have instigated numerous phase II and phase III clinical trials, for example, trials of various mAbs, including those directed against CD52 (alemtuzumab), CD25 (daclizumab), and CD20 (rituximab), and trials of disease-modifying therapies, such as teriflunomide, laquinimod, and fumarate [135, 155]. For example, alemtuzumab, a humanized mAb, targets the surface molecule CD52 on all T-cell populations and other cellular components of the immune system, such as thymocytes, B cells, and monocytes [156].

Offner reported that estrogen and its derivatives exert neuroimmunoprotective effects against EAE and that E2 upregulates the expression of Foxp3 and Ctla4, which contribute to the activity of Tregs, suggesting the therapeutic application of estrogen to MS [157]. Papenfuss et al. also demonstrated that estriol (E3), a pregnancy-specific estrogen, has therapeutic efficacy in MS and EAE and they confirmed that E3 protects mice against EAE by inducing DCs to increase their expression of inhibitory costimulatory markers (PD-L1, PD-L2, B7-H3) and deviate towards a Th2 phenotype [158].

Peroxisome proliferator-activated receptors (PPARs) are members of the nuclear hormone receptor superfamily, which includes receptors for steroids, retinoids, and thyroid hormones, all of which are involved in the immune response [159]. Natarajan et al. demonstrated that PPAR*γ* agonists inhibit EAE by blocking IL12 production, IL12 signaling, and Th1 cell differentiation [160]. Kanakasabai et al. further demonstrated that the PPAR*δ* agonists ameliorate EAE by blocking IFN*γ* and IL17 production by Th1 and Th17 cells. The inhibition of EAE by PPAR*δ* agonists is also associated with reductions in IL12 and IL23 and increases in IL4 and IL10 expression in the CNS and lymphoid organs. This indicates that PPAR*δ* agonists modulate the Th1 and Th17 responses in EAE, and suggests their use in the treatment of MS and other autoimmune diseases [161].

Minocycline, an oral semisynthetic tetracycline antibiotic, can penetrate the CNS and has interesting pleiotropic biological functions and neuroprotective effects, including in demyelinating diseases such as MS [55]. Nikodemova et al. have shown that minocycline attenuates EAE in rats by reducing T-cell infiltration into the spinal cord and downregulating LFA-1 on T cells, but without modifying the production of dominant cytokines [162]. Zabad et al. demonstrated in a cohort study the impact of oral minocycline on clinical and MRI outcomes and serum immune molecules during the 24 months of open-label minocycline treatment. No relapses occurred between months 6 and 24, and the levels of the p40 subunit of IL12 were elevated during the 18 months of treatment, which might have counteracted the proinflammatory effects of IL12R. The downregulation of MMP9 activity was reduced by minocycline treatment [163].

Brines et al. have demonstrated that EPO mediates neuroprotection against experimental ischemic brain injury [164]. Agnello et al. have shown that EPO exerts an anti-inflammatory effect that ameliorates EAE [165]. Yuan et al. also demonstrated that EPO retains its immunomodulatory capacity in both the periphery and the inflamed spinal cord by promoting a massive expansion of Treg cells, inhibiting Th17 polarization and abrogating the proliferation of antigen-presenting DCs [166]. We observed significantly reduced levels of both Th1 and Th17 cells in the CNS and a significantly increased proportion of splenic Tregs in EPO-treated Mog-EAE mice. We also demonstrated that MOG-specific T-cell proliferation was suppressed in the EPO-treated group [167].

The immunomodulatory mechanisms of immunomediated therapeutic agents are not fully understood. Here, we report our current understanding of the immunomodulatory effects of clinically proven and clinically tried agents, and of potential candidate agents, such as decoy receptor 3 (DcR3). We have selectively reviewed their immunomodulation in EAE and MS. Demjen et al. showed that the neutralization of CD95L (FasL) promoted axonal regeneration and functional improvement in an injured animal model, suggesting that this therapeutic strategy may constitute a potent future treatment for human spinal injury [168]. DcR3 is a recognized member of the TNFR superfamily and is predominantly expressed in tumor cells, allowing them to evade immune attack [169]. DcR3 is a soluble receptor that binds to members of the TNF family and can competitively inhibit the binding of TNF to TNFRs [170]. FasL, LIGHT, and TNF-like molecule 1A (TL1A) are all confirmed ligands of DcR3 [171, 172]. When DcR3 binds to FasL, it inhibits FasL-induced apoptosis [169]. It has also recently been shown that DcR3 counteracts the effects of Th17 cells by interfering with FasL-Fas interactions [173]. We have demonstrated that DcR3 ameliorates EAE by directly counteracting inflammation and downregulating Th17 cells in situ [174], implying that DcR3 downregulates the Th17 response and inhibits the inflammation of the CNS in situ during EAE by blocking ligand-receptor interactions, such as Fas-FasL, DR2–LIGHT, and/or DR3–TL1A. Therefore, we introduce DcR3, another immunomodulatory molecule, as a potential candidate for consideration in the clinical treatment of MS.

In summary (Figure 2), these immunomodulatory agents and neuroprotective therapies for MS have great value as clinical agents, to be tested in clinical trials or preclinical studies, and in the development of novel therapeutic strategies for MS [55].

## 8. Concluding Remarks

MS is the most common disabling CNS disease in young adults. It is characterized by recurrent relapses and/or progression, which are attributable to multifocal inflammation, demyelination, and axonal pathology within the brain and/or spinal cord [175]. The effector Th cells play a well-recognized role in the initiation of autoimmune tissue inflammation, and these autoreactive effector CD4^+^ T cells have an established association with the pathogenesis of this disorder [17]. However, in models thought to be driven by Th1 cells, mice lacking the hallmark Th1 cytokine IFN*γ* were not protected from EAE but tended to display enhanced susceptibility to this disease [26]. The identification of Th17 cells has shed light on this apparent discrepancy. Like Th1 cells, polarized Th17 cells have the capacity to cause inflammation and autoimmune disease. A deficiency of the Th17-related cytokine IL23, but not of the Th1-related cytokine IL12, induces resistance to EAE, implying that Th17 cells are the chief contributors to EAE/MS [28], whereas Th1 cells can consistently transfer EAE disease [16, 17]. Komiyama et al. demonstrated that EAE was significantly suppressed in Il17^−/−^ mice, manifested as delayed onset, reduced maximum severity, ameliorated histological changes, and early recovery [176]. However, the outcomes have varied when the differentiation and/or functions of Th17 cells have been blocked in clinical trials of human autoimmune diseases, with notable success only in psoriasis and Crohn's disease, but negative results in relapsing/remitting MS. The strategy of inhibiting the Th17 response has had even less support in preclinical studies in animal models [177].

These data raise the questions of whether MS is mediated solely by Th1 cells or solely by Th17 cells, whether it is mediated by both pathways, or whether perhaps it is mediated by neither pathway [175]. There is growing evidence that autoreactive T cells (particularly Th1 and Th17 cells) participate in the pathophysiology of MS. Although the exact roles of Th1 and Th17 cells in the development of MS lesions are not well understood, it appears that both these effector T-cell populations can cause CNS inflammation and demyelinating lesions in MS and EAE [50, 178].

Our increasing understanding of the immunopathogenic roles of Th1, Th2, and Th17 cells and Tregs in MS/EAE should facilitate the development of novel immunomodulatory therapeutic approaches to MS [179, 180]. The treatment of MS has always been hampered by the untoward adverse effects caused by immunosuppression with agents such as natalizumab [128]. Currently approved disease-modifying treatments achieve their effects primarily by blocking the proinflammatory response in a nonspecific manner. Their limited clinical efficacy calls for a more differentiated and specific therapeutic approach. We can confidently say that IFN*β*, GA, and mitoxantrone are fairly clinically effective for MS patients. The addition of estrogen(s) or minocycline has also shown benefits in the treatment of MS. We have established the protective effects of DcR3 and EPO against EAE [174, 181], but further evidence is required before they can be used clinically for the treatment of MS. More immunomodulatory therapeutic agents are currently in clinical trials, including fingolimod (FTY720), alemtuzumab, and rituximab add-on therapies [182]. The extensive clinical application of these potential novel immunomodulatory therapeutic agents will be under close scrutiny in the near future.

## References

[1] Siffrin V, Brandt AU, Herz J, Zipp F (2007). New insights into adaptive immunity in chronic neuroinflammation. *Advances in Immunology*.

[2] Beeton C, Garcia A, Chandy KG (2007). Induction and clinical scoring of chronic-relapsing experimental autoimmune encephalomyelitis. *Journal of Visualized Experiments*.

[3] Schreiner B, Heppner FL, Becher B (2009). Modeling multiple sclerosis in laboratory animals. *Seminars in Immunopathology*.

[4] Rivers TM, Sprunt DH, Berry GP (1933). Observations on attempts to produce acute disseminated encephalomyelitis in monkeys. *The Journal of Experimental Medicine*.

[5] Steinman L, Zamvil SS (2006). How to successfully apply animal studies in experimental allergic encephalomyelitis to research on multiple sclerosis. *Annals of Neurology*.

[6] Pette M, Fujita K, Kitze B (1990). Myelin basic protein-specific T lymphocyte lines from MS patients and healthy individuals. *Neurology*.

[7] Schluesener HJ, Wekerle H (1985). Autoaggressive T lymphocyte lines recognizing the encephalitogenic region of myelin basic protein: in vitro selection from unprimed rat T lymphocyte populations. *Journal of Immunology*.

[8] Genain CP, Lee-Parritz D, Nguyen MH (1994). In healthy primates, circulating autoreactive T cells mediate autoimmune disease. *Journal of Clinical Investigation*.

[9] Ben-Nun A, Wekerle H, Cohen IR (1981). The rapid isolation of clonable antigen-specific T lymphocyte lines capable of mediating autoimmune encephalomyelitis. *European Journal of Immunology*.

[10] Fort MM, Cheung J, Yen D (2001). IL-25 Induces IL-4, IL-5, and IL-13 and Th2-associated pathologies in vivo. *Immunity*.

[11] Kuchroo VK, Anderson AC, Waldner H, Munder M, Bettelli E, Nicholson LB (2002). T cell response in experimental autoimmune encephalomyelitis (EAE): role of self and cross-reactive antigens in shaping, tuning, and regulating the autopathogenic T cell repertoire. *Annual Review of Immunology*.

[12] Anderson AC, Nicholson LB, Legge KL, Turchin V, Zaghouani H, Kuchroo VK (2000). High frequency of autoreactive myelin proteolipid protein-specific T cells in the periphery of naive mice: mechanisms of selection of the self-reactive repertoire. *Journal of Experimental Medicine*.

[13] Imam SA, Guyton MK, Haque A (2007). Increased calpain correlates with Th1 cytokine profile in PBMCs from MS patients. *Journal of Neuroimmunology*.

[14] Panitch HS, Hirsch RL, Schindler J, Johnson KP (1987). Treatment of multiple sclerosis with gamma interferon: exacerbations associated with activation of the immune system. *Neurology*.

[15] Neukirch F, Lyon-Caen O, Clanet M, Bousquet J, Feingold J, Druet P (1997). Asthma, nasal allergies, and multiple sclerosis. *Journal of Allergy and Clinical Immunology*.

[16] Ando DG, Clayton J, Kono D, Urban JL, Sercarz EE (1989). Encephalitogenic T cells in the B10.PL model of experimental allergic encephalomyelitis (EAE) are of the Th-1 lymphokine subtype. *Cellular Immunology*.

[17] Zamvil SS, Steinman L (1990). The T lymphocyte in experimental allergic encephalomyelitis. *Annual Review of Immunology*.

[18] Yura M, Takahashi I, Serada M (2001). Role of MOG-stimulated th1 type “light up” (GFP+) CD4+ T cells for the development of experimental autoimmune encephalomyelitis (EAE). *Journal of Autoimmunity*.

[19] Mosmann TR, Coffman RL (1989). TH1 and TH2 cells: different patterns of lymphokine secretion lead to different functional properties. *Annual Review of Immunology*.

[20] Adorini L, Guéry JC, Trembleau S (1996). Manipulation of the Th1/Th2 cell balance: an approach to treat human autoimmune diseases?. *Autoimmunity*.

[21] Krakowski M, Owens T (1996). Interferon-*γ* confers resistance to experimental allergic encephalomyelitis. *European Journal of Immunology*.

[22] Tran EH, Prince EN, Owens T (2000). IFN-*γ* shapes immune invasion of the central nervous system via regulation of chemokines. *Journal of Immunology*.

[23] Gran B, Zhang GX, Yu S (2002). IL-12p35-deficient mice are susceptible to experimental autoimmune encephalomyelitis: evidence for redundancy in the IL-12 system in the induction of central nervous system autoimmune demyelination. *Journal of Immunology*.

[24] Zhang GX, Gran B, Yu S (2003). Induction of experimental autoimmune encephalomyelitis in IL-12 receptor-*β*2-deficient mice: IL-12 responsiveness is not required in the pathogenesis of inflammatory demyelination in the central nervous system. *Journal of Immunology*.

[25] Gutcher I, Urich E, Wolter K, Prinz M, Becher B (2006). Interleukin 18-independent engagement of interleukin 18 receptor-*α* is required for autoimmune inflammation. *Nature Immunology*.

[26] Ferber IA, Brocke S, Taylor-Edwards C (1996). Mice with a disrupted IFN-*γ* gene are susceptible to the induction of experimental autoimmune encephalomyelitis (EAE). *Journal of Immunology*.

[27] Oppmann B, Lesley R, Blom B (2000). Novel p19 protein engages IL-12p40 to form a cytokine, IL-23, with biological activities similar as well as distinct from IL-12. *Immunity*.

[28] Cua DJ, Sherlock J, Chen Y (2003). Interleukin-23 rather than interleukin-12 is the critical cytokine for autoimmune inflammation of the brain. *Nature*.

[29] Aggarwal S, Ghilardi N, Xie MH, De Sauvage FJ, Gurney AL (2003). Interleukin-23 promotes a distinct CD4 T cell activation state characterized by the production of interleukin-17. *Journal of Biological Chemistry*.

[30] Langrish CL, Chen Y, Blumenschein WM (2005). IL-23 drives a pathogenic T cell population that induces autoimmune inflammation. *Journal of Experimental Medicine*.

[31] Park H, Li Z, Yang XO (2005). A distinct lineage of CD4 T cells regulates tissue inflammation by producing interleukin 17. *Nature Immunology*.

[32] Harrington LE, Hatton RD, Mangan PR (2005). Interleukin 17-producing CD4+ effector T cells develop via a lineage distinct from the T helper type 1 and 2 lineages. *Nature Immunology*.

[33] Dong C (2006). Diversification of T-helper-cell lineages: finding the family root of IL-17-producing cells. *Nature Reviews Immunology*.

[34] Miossec P, Korn T, Kuchroo VK (2009). Interleukin-17 and type 17 helper T cells. *The New England Journal of Medicine*.

[35] Annunziato F, Cosmi L, Santarlasci V (2007). Phenotypic and functional features of human Th17 cells. *Journal of Experimental Medicine*.

[36] Hedegaard CJ, Krakauer M, Bendtzen K, Lund H, Sellebjerg F, Nielsen CH (2008). T helper cell type 1 (Th1), Th2 and Th17 responses to myelin basic protein and disease activity in multiple sclerosis. *Immunology*.

[37] Parham C, Chirica M, Timans J (2002). A receptor for the heterodimeric cytokine IL-23 is composed of IL-12R*β*1 and a novel cytokine receptor subunit, IL-23R. *Journal of Immunology*.

[38] Mangan PR, Harrington LE, O’Quinn DB (2006). Transforming growth factor-*β* induces development of the T H17 lineage. *Nature*.

[39] Bettelli E, Carrier Y, Gao W (2006). Reciprocal developmental pathways for the generation of pathogenic effector TH17 and regulatory T cells. *Nature*.

[40] Yang L, Anderson DE, Baecher-Allan C (2008). IL-21 and TGF-*β* are required for differentiation of human T H17 cells. *Nature*.

[41] Korn T, Bettelli E, Gao W (2007). IL-21 initiates an alternative pathway to induce proinflammatory T H17 cells. *Nature*.

[42] Lock C, Hermans G, Pedotti R (2002). Gene-microarray analysis of multiple sclerosis lesions yields new targets validated in autoimmune encephalomyelitis. *Nature Medicine*.

[43] Korn T, Bettelli E, Oukka M, Kuchroo VK (2009). IL-17 and Th17 cells. *Annual Review of Immunology*.

[44] Chen Z, O’Shea JJ (2008). Th17 cells: a new fate for differentiating helper T cells. *Immunologic Research*.

[45] Lovett-Racke AE, Rocchini AE, Choy J (2004). Silencing T-bet defines a critical role in the differentiation of autoreactive T lymphocytes. *Immunity*.

[46] Ouyang W, Ranganath SH, Weindel K (1998). Inhibition of Th1 development mediated by GATA-3 through an IL-4- independent mechanism. *Immunity*.

[47] Ouyang W, Löhning M, Gao Z (2000). Stat6-independent GATA-3 autoactivation directs IL-4-independent Th2 development and commitment. *Immunity*.

[48] Bettelli E, Sullivan B, Szabo SJ, Sobel RA, Glimcher LH, Kuchroo VK (2004). Loss of T-bet, but not STAT1, prevents the development of experimental autoimmune encephalomyelitis. *Journal of Experimental Medicine*.

[49] O’Connor RA, Prendergast CT, Sabatos CA (2008). Cutting edge: Th1 cells facilitate the entry of Th17 cells to the central nervous system during experimental autoimmune encephalomyelitis. *Journal of Immunology*.

[50] Edwards LJ, Robins RA, Constantinescu CS (2010). Th17/Th1 phenotype in demyelinating disease. *Cytokine*.

[51] Pai SY, Truitt ML, Ho IC (2004). GATA-3 deficiency abrogates the development and maintenance of T helper type 2 cells. *Proceedings of the National Academy of Sciences of the United States of America*.

[52] Ivanov II, McKenzie BS, Zhou L (2006). The orphan nuclear receptor ROR*γ*t directs the differentiation program of proinflammatory IL-17+ T helper cells. *Cell*.

[53] Fontenot JD, Gavin MA, Rudensky AY (2003). Foxp3 programs the development and function of CD4+CD25+ regulatory T cells. *Nature Immunology*.

[54] Hirota K, Martin B, Veldhoen M (2010). Development, regulation and functional capacities of Th17 cells. *Seminars in Immunopathology*.

[55] Lopez-Diego RS, Weiner HL (2008). Novel therapeutic strategies for multiple sclerosis—a multifaceted adversary. *Nature Reviews Drug Discovery*.

[56] Matusevicius D, Kivisäkk P, He B (1999). Interleukin-17 mRNA expression in blood and CSF mononuclear cells is augmented in multiple sclerosis. *Multiple Sclerosis*.

[57] Ransohoff RM, Kivisäkk P, Kidd G (2003). Three or more routes for leukocyte migration into the central nervous system. *Nature Reviews Immunology*.

[58] Xiao BG, Diab A, Zhu J, Van Der Meide P, Link H (1998). Astrocytes induce hyporesponses of myelin basic protein-reactive T and B cell function. *Journal of Neuroimmunology*.

[59] Dijkstra CD, De Groot CJA, Huitinga I (1992). The role of macrophages in demyelination. *Journal of Neuroimmunology*.

[60] Kasper LH, Shoemaker J (2010). Multiple sclerosis immunology: the healthy immune system vs the MS immune system. *Neurology*.

[61] Deshpande P, King IL, Segal BM (2007). Cutting edge: CNS CD11c+ cells, from mice with encephalomyelitis polarize Th17 cells, and support CD25+CD4+ T cell-mediated immunosuppression, suggesting dual roles in the disease process. *Journal of Immunology*.

[62] Cserr HF, Knopf PM (1992). Cervical lymphatics, the blood-brain barrier and the immunoreactivity of the brain: a new view. *Immunology Today*.

[63] Hemmer B, Nessler S, Zhou D, Kieseier B, Hartung HP (2006). Immunopathogenesis and immunotherapy of multiple sclerosis. *Nature Clinical Practice Neurology*.

[64] Neumann H, Cavalie A, Jenne DE, Wekerle H (1995). Induction of MHC class I genes in neurons. *Science*.

[65] Dandekar AA, Wu GF, Pewe L, Perlman S (2001). Axonal damage is T cell mediated and occurs concomitantly with demyelination in mice infected with a neurotropic coronavirus. *Journal of Virology*.

[66] Probert L, Eugster HP, Akassoglu K (2000). TNFR1 signalling is critical for the development of demyelination and the limitation of T-cell responses during immune-mediated CNS disease. *Brain*.

[67] Tzartos JS, Friese MA, Craner MJ (2008). Interleukin-17 production in central nervous system-infiltrating T cells and glial cells is associated with active disease in multiple sclerosis. *American Journal of Pathology*.

[68] Crome SQ, Wang AY, Kang CY, Levings MK (2009). The role of retinoic acid-related orphan receptor variant 2 and IL-17 in the development and function of human CD4+ T cells. *European Journal of Immunology*.

[69] Li Y, Chu N, Hu A, Gran B, Rostami A, Zhang GX (2007). Increased IL-23p19 expression in multiple sclerosis lesions and its induction in microglia. *Brain*.

[70] Kebir H, Kreymborg K, Ifergan I (2007). Human TH17 lymphocytes promote blood-brain barrier disruption and central nervous system inflammation. *Nature Medicine*.

[71] Linker RA, Lühder F, Kallen KJ (2008). IL-6 transsignalling modulates the early effector phase of EAE and targets the blood-brain barrier. *Journal of Neuroimmunology*.

[72] Ifergan I, Kébir H, Bernard M (2008). The blood-brain barrier induces differentiation of migrating monocytes into Th17-polarizing dendritic cells. *Brain*.

[73] Miljkovic D, Momcilovic M, Stojanovic I, Stosic-Grujicic S, Ramic Z, Mostarica-Stojkovic M (2007). Astrocytes stimulate interleukin-17 and interferon-*γ* production in vitro. *Journal of Neuroscience Research*.

[74] Das Sarma J, Ciric B, Marek R (2009). Functional interleukin-17 receptor A is expressed in central nervous system glia and upregulated in experimental autoimmune encephalomyelitis. *Journal of Neuroinflammation*.

[75] Ma X, Reynolds SL, Baker BJ, Li X, Benveniste EN, Qin H (2010). IL-17 enhancement of the IL-6 signaling cascade in astrocytes. *Journal of Immunology*.

[76] Kang Z, Altuntas CZ, Gulen MF (2010). Astrocyte-restricted ablation of interleukin-17-induced act1-mediated signaling ameliorates autoimmune encephalomyelitis. *Immunity*.

[77] Merkler D, Böscke R, Schmelting B (2006). Differential macrophage/microglia activation in neocortical EAE lesions in the marmoset monkey. *Brain Pathology*.

[78] Lees JR, Iwakura Y, Russell JH (2008). Host T cells are the main producers of IL-17 within the central nervous system during initiation of experimental autoimmune encephalomyelitis induced by adoptive transfer of Th1 cell lines. *Journal of Immunology*.

[79] Sutton C, Brereton C, Keogh B, Mills KHG, Lavelle EC (2006). A crucial role for interleukin (IL)-1 in the induction of IL-17-producing T cells that mediate autoimmune encephalomyelitis. *Journal of Experimental Medicine*.

[80] De Jong BA, Huizinga TWJ, Bollen ELEM (2002). Production of IL-1*β* and IL-1Ra as risk factors for susceptibility and progression of relapse-onset multiple sclerosis. *Journal of Neuroimmunology*.

[81] Broberg EK, Salmi AA, Hukkanen V (2004). IL-4 is the key regulator in herpes simplex virus-based gene therapy of BALB/c experimental autoimmune encephalomyelitis. *Neuroscience Letters*.

[82] Haas J, Hug A, Viehöver A (2005). Reduced suppressive effect of CD4+CD25high regulatory T cells on the T cell immune response against myelin oligodendrocyte glycoprotein in patients with multiple sclerosis. *European Journal of Immunology*.

[83] Singh SP, Zhang HH, Foley JF, Hedrick MN, Farber JM (2008). Human T cells that are able to produce IL-17 express the chemokine receptor CCR6. *Journal of Immunology*.

[84] Haak S, Croxford AL, Kreymborg K (2009). IL-17A and IL-17F do not contribute vitally to autoimmune neuro-inflammation in mice. *Journal of Clinical Investigation*.

[85] Liu X, Yun SL, Yu CR, Egwuagu CE (2008). Loss of STAT3 in CD4+ T cells prevents development of experimental autoimmune diseases. *Journal of Immunology*.

[86] Stromnes IM, Cerretti LM, Liggitt D, Harris RA, Goverman JM (2008). Differential regulation of central nervous system autoimmunity by T H1 and TH17 cells. *Nature Medicine*.

[87] Zhou L, Ivanov II, Spolski R (2007). IL-6 programs TH-17 cell differentiation by promoting sequential engagement of the IL-21 and IL-23 pathways. *Nature Immunology*.

[88] Zhang Z, Rosenbaum JT, Zhong W, Lim C, Hinrichs DJ (2010). Costimulation of Th17 cells: adding fuel or putting out the fire in the inflamed gut?. *Seminars in Immunopathology*.

[89] Vollmer TL, Liu R, Price M, Rhodes S, La Cava A, Shi FD (2005). Differential effects of IL-21 during initiation and progression of autoimmunity against neuroantigen. *Journal of Immunology*.

[90] Pot C, Jin H, Awasthi A (2009). Cutting edge: IL-27 induces the transcription factor c-Maf, cytokine IL-21, and the costimulatory receptor ICOS that coordinately act together to promote differentiation of IL-10-producing Tr1 cells. *Journal of Immunology*.

[91] Fitzgerald DC, Ciric B, Touil T (2007). Suppressive effect of IL-27 on encephalitogenic Th17 cells and the effector phase of experimental autoimmune encephalomyelitis. *Journal of Immunology*.

[92] Jäger A, Dardalhon V, Sobel RA, Bettelli E, Kuchroo VK (2009). Th1, Th17, and Th9 effector cells induce experimental autoimmune encephalomyelitis with different pathological phenotypes. *Journal of Immunology*.

[93] Mueller AM, Pedré X, Killian S, David M, Steinbrecher A (2009). The Decoy Receptor 3 (DcR3, TNFRSF6B) suppresses Th17 immune responses and is abundant in human cerebrospinal fluid. *Journal of Neuroimmunology*.

[94] Nowak EC, Weaver CT, Turner H (2009). IL-9 as a mediator of Th17-driven inflammatory disease. *Journal of Experimental Medicine*.

[95] Wiendl H, Toyka KV, Rieckmann P (2008). Basic and escalating immunomodulatory treatments in multiple sclerosis: current therapeutic recommendations. *Journal of Neurology*.

[96] Goodin DS, Frohman EM, Garmany GP (2002). Disease modifying therapies in multiple sclerosis: report of the therapeutics and technology assessment subcommittee of the American academy of neurology and the MS council for clinical practice guidelines. *Neurology*.

[97] Henze T, Rieckmann P, Toyka KV (2006). Symptomatic treatment of multiple sclerosis: multiple Sclerosis Therapy Consensus Group (MSTCG) of the German Multiple Sclerosis Society. *European Neurology*.

[98] Hemmer B, Hartung HP (2007). Toward the development of rational therapies in multiple sclerosis: what is on the horizon?. *Annals of Neurology*.

[99] Bielekova B, Becker BL (2010). Monoclonal antibodies in MS: mechanisms of action. *Neurology*.

[100] Bates D (2009). Alemtuzumab. *International MS journal/MS Forum*.

[101] Hawker K, O’Connor P, Freedman MS (2009). Rituximab in patients with primary progressive multiple sclerosis: results of a randomized double-blind placebo-controlled multicenter trial. *Annals of Neurology*.

[102] Bielekova B, Howard T, Packer AN (2009). Effect of anti-CD25 antibody daclizumab in the inhibition of inflammation and stabilization of disease progression in multiple sclerosis. *Archives of Neurology*.

[103] Dhib-Jalbut S (2002). Mechanisms of action of interferons and glatiramer acetate in multiple sclerosis. *Neurology*.

[104] Krakauer M, Sorensen P, Khademi M, Olsson T, Sellebjerg F (2008). Increased IL-10 mRNA and IL-23 mRNA expression in multiple sclerosis: interferon-*β* treatment increases IL-10 mRNA expression while reducing IL-23 mRNA expression. *Multiple Sclerosis*.

[105] Ramgolam VS, Sha Y, Jin J, Zhang X, Markovic-Plese S (2009). IFN-*β* inhibits human Th17 cell differentiation. *Journal of Immunology*.

[106] Guo B, Chang EY, Cheng G (2008). The type I IFN induction pathway constrains Th17-mediated autoimmune inflammation in mice. *Journal of Clinical Investigation*.

[107] Sweeney CM, Lonergan R, Basdeo SA (2011). IL-27 mediates the response to IFN-*β* therapy in multiple sclerosis patients by inhibiting Th17 cells. *Brain, Behavior and Immunity*.

[108] Galligan CL, Pennell LM, Murooka TT (2010). Interferon-*β* is a key regulator of proinflammatory events in experimental autoimmune encephalomyelitis. *Multiple Sclerosis*.

[109] Axtell RC, De Jong BA, Boniface K (2010). T helper type 1 and 17 cells determine efficacy of interferon-*β* in multiple sclerosis and experimental encephalomyelitis. *Nature Medicine*.

[110] Axtell RC, Raman C, Steinman L (2011). Interferon-*β* exacerbates Th17-mediated inflammatory disease. *Trends in Immunology*.

[111] Antel J, Bar-Or A (2006). Roles of immunoglobulins and B cells in multiple sclerosis: from pathogenesis to treatment. *Journal of Neuroimmunology*.

[112] Fillatreau S, Gray D, Anderton SM (2008). Not always the bad guys: B cells as regulators of autoimmune pathology. *Nature Reviews Immunology*.

[113] Ramgolam VS, Sha Y, Marcus KL (2011). B cells as a therapeutic target for IFN-*β* in relapsing-remitting multiple sclerosis. *Journal of Immunology*.

[114] Valenzuela RM, Costello K, Chen M, Said A, Johnson KP, Dhib-Jalbut S (2007). Clinical response to glatiramer acetate correlates with modulation of IFN-*γ* and IL-4 expression in multiple sclerosis. *Multiple Sclerosis*.

[115] Saresella M, Marventano I, Longhi R (2008). CD4+CD25+FoxP3+PD1- Regulatory T cells in acute and stable relapsing-remitting multiple sclerosis and their modulation by therapy. *FASEB Journal*.

[116] Hong J, Li N, Zhang X, Zheng B, Zhang JZ (2005). Induction of CD4+CD25+ regulatory T cells by copolymer-I through activation of transcription factor Foxp3. *Proceedings of the National Academy of Sciences of the United States of America*.

[117] Vieira PL, Heystek HC, Wormmeester J, Wierenga EA, Kapsenberg ML (2003). Glatiramer acetate (copolymer-1, copaxone) promotes Th2 cell development and increased IL-10 production through modulation of dendritic cells. *Journal of Immunology*.

[118] Jung S, Siglienti I, Grauer O, Magnus T, Scarlato G, Toyka K (2004). Induction of IL-10 in rat peritoneal macrophages and dendritic cells by glatiramer acetate. *Journal of Neuroimmunology*.

[119] Elovaara I, Ukkonen M, Leppäkynnäs M (2000). Adhesion molecules in multiple sclerosis: relation to subtypes of disease and methylprednisolone therapy. *Archives of Neurology*.

[120] Elovaara I, Lällä M, Spare E, Lehtimäki T, Dastidar P (1998). Methylprednisolone reduces adhesion molecules in blood and cerebrospinal fluid in patients with MS. *Neurology*.

[121] Theien BE, Vanderlugt CL, Eagar TN (2001). Discordant effects of anti-VLA-4 treatment before and after onset of relapsing experimental autoimmune encephalomyelitis. *Journal of Clinical Investigation*.

[122] Stüve O, Marra CM, Jerome KR (2006). Immune surveillance in multiple sclerosis patients treated with natalizumab. *Annals of Neurology*.

[123] Langer-Gould A, Atlas SW, Green AJ, Bollen AW, Pelletier D (2005). Progressive multifocal leukoencephalopathy in a patient treated with natalizumab. *The New England Journal of Medicine*.

[124] Kleinschmidt-DeMasters BK, Tyler KL (2005). Progressive multifocal leukoencephalopathy complicating treatment with natalizumab and interferon *β*-1a for multiple sclerosis. *The New England Journal of Medicine*.

[125] Hellwig K, Gold R (2011). Progressive multifocal leukoencephalopathy and natalizumab. *Journal of Neurology*.

[126] Gorelik L, Lerner M, Bixler S (2010). Anti-JC virus antibodies: implications for PML risk stratification. *Annals of Neurology*.

[127] Richard R (2011). New risk data on PML puts hard numbers on antibody status, immunosuppressants, and treatment duration. *Neurology Today*.

[128] Warnke C, Menge T, Hartung HP (2010). Natalizumab and progressive multifocal leukoencephalopathy: what are the causal factors and can it be avoided?. *Archives of Neurology*.

[129] Hartung HP, Gonsette R, König N (2002). Mitoxantrone in progressive multiple sclerosis: a placebo-controlled, double-blind, randomised, multicentre trial. *The Lancet*.

[130] Vogelgesang A, Rosenberg S, Skrzipek S, Bröker BM, Dressel A (2010). Mitoxantrone treatment in multiple sclerosis induces TH2-type cytokines. *Acta Neurologica Scandinavica*.

[131] Hohlfeld R (2011). Multiple sclerosis: cladribine-a contentious therapeutic contender for MS. *Nature Reviews Neurology*.

[132] Matloubian M, Lo CG, Cinamon G (2004). Lymphocyte egress from thymus and peripheral lymphoid organs is dependent on S1P receptor 1. *Nature*.

[133] Ehling R, Berger T, Reindl M (2010). Multiple sclerosis—established and novel therapeutic approaches. *Central Nervous System Agents in Medicinal Chemistry*.

[134] Strader CR, Pearce CJ, Oberlies NH (2011). Fingolimod (FTY720): a recently approved multiple sclerosis drug based on a fungal secondary metabolite. *Journal of Natural Products*.

[135] Lim SY, Constantinescu CS (2010). Current and future disease-modifying therapies in multiple sclerosis. *International Journal of Clinical Practice*.

[136] Harada J, Foley M, Moskowitz MA, Waeber C (2004). Sphingosine-1-phosphate induces proliferation and morphological changes of neural progenitor cells. *Journal of Neurochemistry*.

[137] Allende ML, Proia RL (2002). Sphingosine-1-phosphate receptors and the development of the vascular system. *Biochimica et Biophysica Acta*.

[138] Jolly PS, Bektas M, Olivera A (2004). Transactivation of sphingosine-1-phosphate receptors by Fc*ε*RI triggering is required for normal mast cell degranulation and chemotaxis. *Journal of Experimental Medicine*.

[139] Donati C, Meacci E, Nuti F, Becciolini L, Farnararo M, Bruni P (2005). Sphingosine 1-phosphate regulates myogenic differentiation: a major role for S1P2 receptor. *FASEB Journal*.

[140] Krump-Konvalinkova V, Yasuda S, Rubic T (2005). Stable knock-down of the sphingosine 1-phosphate receptor S1P1 influences multiple functions of human endothelial cells. *Arteriosclerosis, Thrombosis, and Vascular Biology*.

[141] Halin C, Scimone ML, Bonasio R (2005). The S1P-analog FTY720 differentially modulates T-cell homing via HEV: T-cell-expressed S1P1 amplifies integrin activation in peripheral lymph nodes but not in Peyer patches. *Blood*.

[142] Tao R, Hoover HE, Zhang J, Honbo N, Alano CC, Karliner JS (2009). Cardiomyocyte S1P1 receptor-mediated extracellular signal-related kinase signaling and desensitization. *Journal of Cardiovascular Pharmacology*.

[143] Brinkmann V, Davis MD, Heise CE (2002). The immune modulator FTY720 targets sphingosine 1-phosphate receptors. *Journal of Biological Chemistry*.

[144] Kataoka H, Sugahara K, Shimano K (2005). FTY720, sphingosine 1-phosphate receptor modulator, ameliorates experimental autoimmune encephalomyelitis by inhibition of T cell infiltration. *Cellular &amp; Molecular Immunology*.

[145] Brinkmann V (2009). FTY720 (fingolimod) in Multiple Sclerosis: therapeutic effects in the immune and the central nervous system. *British Journal of Pharmacology*.

[146] Mehling M, Lindberg R, Raulf F (2010). Th17 central memory T cells are reduced by FTY720 in patients with multiple sclerosis. *Neurology*.

[147] Webb M, Tham CS, Lin FF (2004). Sphingosine 1-phosphate receptor agonists attenuate relapsing-remitting experimental autoimmune encephalitis in SJL mice. *Journal of Neuroimmunology*.

[148] Van Doorn R, Van Horssen J, Verzijl D (2010). Sphingosine 1-phosphate receptor 1 and 3 are upregulated in multiple sclerosis lesions. *GLIA*.

[149] Rouach N, Pébay A, Même W (2006). S1P inhibits gap junctions in astrocytes: involvement of G and Rho GTPase/ROCK. *European Journal of Neuroscience*.

[150] Beutler E (1992). Cladribine (2-chlorodeoxyadenosine). *The Lancet*.

[151] Carson DA, Wasson DB, Taetle R, Yu A (1983). Specific toxicity of 2-chlorodeoxyadenosine toward resting and proliferating human lymphocytes. *Blood*.

[152] Yates D (2010). Multiple sclerosis: orally administered cladribine displays efficacy in multiple sclerosis trial. *Nature Reviews Neurology*.

[153] Giovannoni G, Cook S, Rammohan K (2011). Sustained disease-activity-free status in patients with relapsing-remitting multiple sclerosis treated with cladribine tablets in the CLARITY study: a post-hoc and subgroup analysis. *The Lancet Neurology*.

[154] Giovannoni G, Comi G, Cook S (2010). A placebo-controlled trial of oral cladribine for relapsing multiple sclerosis. *The New England Journal of Medicine*.

[155] Barten LJ, Allington DR, Procacci KA, Rivey MP (2010). New approaches in the management of multiple sclerosis. *Drug Design, Development and Therapy*.

[156] Minagar A, Alexander JS, Sahraian MA, Zivadinov R (2010). Alemtuzumab and multiple sclerosis: therapeutic application. *Expert Opinion on Biological Therapy*.

[157] Offner H (2004). Neuroimmunoprotective effects of estrogen and derivatives in experimental autoimmune encephalomyelitis: therapeutic implications for multiple sclerosis. *Journal of Neuroscience Research*.

[158] Papenfuss TL, Powell ND, McClain MA (2011). Estriol generates tolerogenic dendritic cells in vivo that protect against autoimmunity. *Journal of Immunology*.

[159] Dinarello CA (2010). Anti-inflammatory agents: present and future. *Cell*.

[160] Natarajan C, Bright JJ (2002). Peroxisome proliferator-activated receptor-gamma agonist inhibit experimental allergic encephalomyelitis by blocking IL-12 production, IL-12 signaling and Th1 differentiation. *Genes and Immunity*.

[161] Kanakasabai S, Chearwae W, Walline CC, Iams W, Adams SM, Bright JJ (2010). Peroxisome proliferator-activated receptor *δ* agonists inhibit T helper type 1 (Th1) and Th17 responses in experimental allergic encephalomyelitis. *Immunology*.

[162] Nikodemova M, Lee J, Fabry Z, Duncan ID (2010). Minocycline attenuates experimental autoimmune encephalomyelitis in rats by reducing T cell infiltration into the spinal cord. *Journal of Neuroimmunology*.

[163] Zabad RK, Metz LM, Todoruk TR (2007). The clinical response to minocycline in multiple sclerosis is accompanied by beneficial immune changes: a pilot study. *Multiple Sclerosis*.

[164] Brines ML, Ghezzi P, Keenan S (2000). Erythropoietin crosses the blood-brain barrier to protect against experimental brain injury. *Proceedings of the National Academy of Sciences of the United States of America*.

[165] Agnello D, Bigini P, Villa P (2002). Erythropoietin exerts an anti-inflammatory effect on the CNS in a model of experimental autoimmune encephalomyelitis. *Brain Research*.

[166] Yuan R, Maeda Y, Li W, Lu W, Cook S, Dowling P (2008). Erythropoietin: a potent inducer of peripheral immuno/inflammatory modulation in autoimmune EAE. *PLoS ONE*.

[167] Chen SJ, Wang YL, Lo WT (2010). Erythropoietin enhances endogenous haem oxygenase-1 and represses immune responses to ameliorate experimental autoimmune encephalomyelitis. *Clinical and Experimental Immunology*.

[168] Demjen D, Klussmann S, Kleber S (2004). Neutralization of CD95 ligand promotes regeneration and functional recovery after spinal cord injury. *Nature Medicine*.

[169] Pitti RM, Marsters SA, Lawrence DA (1998). Genomic amplification of a decoy receptor for Fas ligand in lung and colon cancer. *Nature*.

[170] Hayashi S, Miura Y, Nishiyama T (2007). Decoy receptor 3 expressed in rheumatoid synovial fibroblasts protects the cells against fas-induced apoptosis. *Arthritis and Rheumatism*.

[171] Zhang J, Salcedo TW, Wan X (2001). Modulation of T-cell responses to alloantigens by TR6/DcR3. *Journal of Clinical Investigation*.

[172] Yu KY, Kwon B, Ni J, Zhai Y, Ebner R, Kwon BS (1999). A newly identified member of tumor necrosis factor receptor superfamily (TR6) suppresses LIGHT-mediated apoptosis. *Journal of Biological Chemistry*.

[173] Sabelko-Downes KA, Cross AH, Russell JH (1999). Dual role for Fas ligand in the initiation of and recovery from experimental allergic encephalomyelitis. *Journal of Experimental Medicine*.

[174] Chen SJ, Wang YL, Kao JH (2009). Decoy receptor 3 ameliorates experimental autoimmune encephalomyelitis by directly counteracting local inflammation and downregulating Th17 cells. *Molecular Immunology*.

[175] Korn T (2008). Pathophysiology of multiple sclerosis. *Journal of Neurology*.

[176] Komiyama Y, Nakae S, Matsuki T (2006). IL-17 plays an important role in the development of experimental autoimmune encephalomyelitis. *Journal of Immunology*.

[177] Steinman L (2010). Mixed results with modulation of T H-17 cells in human autoimmune diseases. *Nature Immunology*.

[178] Murphy AC, Lalor SJ, Lynch MA, Mills KHG (2010). Infiltration of Th1 and Th17 cells and activation of microglia in the CNS during the course of experimental autoimmune encephalomyelitis. *Brain, Behavior, and Immunity*.

[179] Fletcher JM, Lalor SJ, Sweeney CM, Tubridy N, Mills KHG (2010). T cells in multiple sclerosis and experimental autoimmune encephalomyelitis. *Clinical and Experimental Immunology*.

[180] Lovett-Racke AE, Yang Y, Racke MK (2011). Th1 versus Th17: are T cell cytokines relevant in multiple sclerosis?. *Biochimica et Biophysica Acta*.

[181] Wang YL, Chou FC, Sung HH (2010). Decoy receptor 3 protects non-obese diabetic mice from autoimmune diabetes by regulating dendritic cell maturation and function. *Molecular Immunology*.

[182] Naismith RT, Piccio L, Lyons JA (2010). Rituximab add-on therapy for breakthrough relapsing multiple sclerosis: a 52-week phase II trial. *Neurology*.

